# Medical treatment of dystonia

**DOI:** 10.1186/s40734-016-0047-6

**Published:** 2016-12-19

**Authors:** Pichet Termsarasab, Thananan Thammongkolchai, Steven J. Frucht

**Affiliations:** 1 Movement Disorder Division, Department of Neurology, Icahn School of Medicine at Mount Sinai, New York, USA; 2 Department of Neurology, University Hospitals Cleveland Medical Center, Cleveland, USA

**Keywords:** Dystonia, Treatment, Medications, Anticholinergic, Baclofen, Clonazepam, Pharmacology

## Abstract

Therapeutic strategies in dystonia have evolved considerably in the past few decades. Three major treatment modalities include oral medications, botulinum toxin injections and surgical therapies, particularly deep brain stimulation. Although there has been a tremendous interest in the later two modalities, there are relatively few recent reviews of oral treatment. We review the medical treatment of dystonia, focusing on three major neurotransmitter systems: cholinergic, GABAergic and dopaminergic. We also provide a practical guide to medication selection, therapeutic strategy and unmet needs.

## Introduction

Three main approaches are employed in the treatment of dystonia: pharmacological therapies, botulinum toxin injection (BoNT) and surgical interventions. The current review focuses **only** on medical therapy, as this area is less commonly addressed in the literature. Four major categories of medications are most commonly used: anticholinergics (particularly trihexyphenidyl), baclofen, benzodiazepines (particularly clonazepam), and dopamine-related medications. We suggest the mnemonic “ABCD”, which stands for **A**nticholinergics or **A**rtane®, **B**aclofen, **C**lonazepam, and **D**opamine-related medications as a helpful way to remember these options. Medical therapy in dystonia is largely empiric, and at times may seem anecdotal.

## Review

### Neurotransmitter systems critical to medical treatment in dystonia

**Fig. 1:**
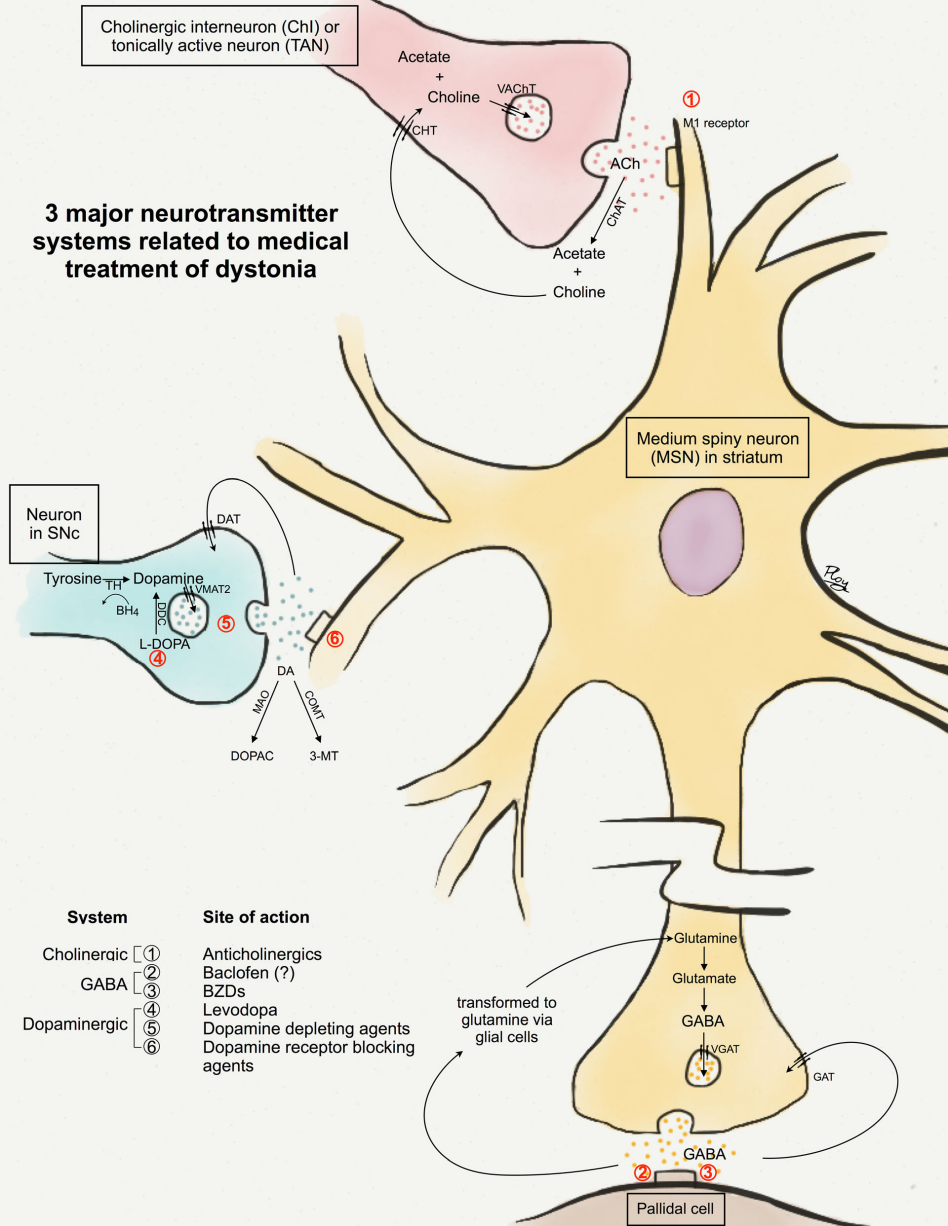
The three major neurotransmitters in dystonia. This figure illustrates the three neurotransmitters in the striatum (cholinergic [*in pink*], GABAergic [*in yellow and brown*] and dopaminergic [*in blue*]), their processes at synaptic levels and affected targets. Of note, other neurotransmitters such as cannabinoids and serotonin may also play a role in dystonia but are not shown here. **1) Cholinergic system.** Giant asypiny or cholinergic interneurons (ChIs; *in pink*), also referred to as tonically active neurons (TANs), are a main cholinergic input to medium spiny neurons (MSNs; *in yellow*) in the striatum. At the synaptic level, ACh is synthesized in presynaptic terminals by acetylation of choline, catalyzed by the enzyme choline acetyltransferase (ChAT). ACh is then transported into vesicles by the vesicular ACh transporter (VAChT). After ACh is released at synaptic clefts, it binds to muscarinic (M1-4 subtypes) and/or nicotinic receptors in order to have further action downstream. The remaining ACh at the synaptic cleft is subsequently metabolized by acetylcholinesterase (AChE) into acetate and choline. The latter is taken up into the presynaptic terminal by the choline transporter (CHT). **2) GABAergic system.** GABA is present widely in neurons subserving basal ganglia circuitry including the MSNs, and both internal and external segments of the globus pallidus. In this figure, only the synapse between the MSN and the pallidal cell (*in brown*) is demonstrated. At the synaptic level, GABA is synthesized from glutamate in presynaptic terminals. It is then packed into vesicles via the vesicular GABA transporter (VGAT) before being released into synaptic clefts. GABA subsequently binds to postsynaptic receptors. The remaining GABA at the synaptic clefts is transported back to presynaptic terminals by two methods: 1) direct reuptake by GABA transporters (GAT) at presynaptic terminals 2) indirect transport via adjacent glial cells requiring transformation to glutamine prior to returning to presynaptic terminals. **3) Dopaminergic system. **The MSNs also receive dopaminergic input from neurons in the substantia nigra pars compacta (SNc) via the nigrostriatal pathway (*in blue*). At the synaptic level, dopamine is synthesized in presynaptic terminals from tyrosine by the enzyme tyrosine hydroxylase (TH) requiring tetrahydrobiopterin (BH_4_) as a cofactor. Dopamine (DA) and other monoamines are packaged into vesicles in presynaptic terminals by the enzyme vesicular monoamine transporter 2 (VMAT2). The monoamines are then released to synaptic clefts and bind to postsynaptic receptors including dopamine receptors (D1-5). Dopamine at synaptic clefts is degraded by the enzymes monoamine oxidase (MAO) and cathechol-*O*-methyl transferase (COMT) into 3,4-dihydroxyphenylacetic acid (DOPAC) and 3-methoxytyramine (3-MT) respectively. The remaining dopamine is subsequently transported back to presynaptic terminals by the dopamine transporters (DAT). The prototypic medications affecting each neurotransmitter systems and their sites of action are listed at the left lower corner. *Anticholinergics* act postsynaptically as muscarinic receptor antagonists, particularly at M1 receptors. *Baclofen* is a GABA_B_ receptor agonist. In the spinal cord, it acts at both presynaptic (excitatory glutamatergic neurons) and postsynaptic (of inhibitory interneurons) terminals. However, its sites of action in the basal ganglia (presynaptic vs. postsynaptic or both) remain unclear (shown as “?”). *Benzodiazepines (BZDs)* bind to GABA_A_ receptors, leading to increased frequency of chloride channel opening and thereby inhibitory signals. Levodopa (L-DOPA) is converted to dopamine in presynaptic terminals by the enzyme DOPA decarboxylase (DDC). *Dopamine depleting agents* such as tetrabenazine (TBZ) acts at presynaptic terminals by inhibiting the VMAT2 enzyme which then impairs dopamine transport into vesicles. *Dopamine receptor blocking agents (DRBAs)*, in contrast, acts postsynaptically by blocking dopamine receptors

Three main neurotransmitter systems are involved: cholinergic, GABAergic and dopaminergic systems. We will consider each system separately (Fig. 1).

#### Cholinergic system

Giant aspiny interneurons or cholinergic interneurons (ChIs) serve as an intrinsic source of acetylcholine (ACh) to the medium spiny neurons (MSNs) in the striatum, whereas pedunculopontine nucleus neurons serve as an extrinsic source. ChIs comprise only 1–3% of all striatal cells, but provide a main source of ACh to the MSNs. They are also referred to as tonically active neurons, given characteristic property of autonomous firing without synaptic activity [1]. Hyperactivity of the ChIs may explain improvement of dystonia with anticholinergics [2]. More recent evidence has also supported the role of ChIs in abnormal corticostriatal synaptic plasticity [3].

Several anticholinergics including trihexyphenidyl, benztropine, ethopropazine, procyclidine and biperiden have been used in dystonia [4–8]. Trihexyphenidyl is the most commonly employed medication. Benztropine is less frequently used, whereas the others are infrequently used in current clinical practice. Generally the anticholinergics act as antagonists at postsynaptic M1 receptors. Some medications also act at other receptors, e.g. biperiden at nicotinic receptors, and procyclidine at M2 and M4 receptors.

#### GABAergic system

GABA is an inhibitory neurotransmitter in the brain and spinal cord. In addition to the MSNs, GABA is present widely in neurons subserving basal ganglia circuitry. The role of GABA in dystonia pathophysiology remains unclear. One study showed abnormal GABA_A_ receptor binding in motor cortices in primary dystonia, probably leading to sensorimotor disinhibition [9]; another study found no change in focal hand dystonia [10].

As a muscle relaxant, baclofen is an agonist of GABA_B_ receptors at presynaptic terminals of excitatory glutamatergic neurons, and at postsynaptic sites of inhibitory interneurons in the spinal cord [11]. Its mechanism in dystonia is less understood. Baclofen is generally considered to be less effective than anticholinergics for dystonia [6].

Benzodiazepines are also medications primarily affecting the GABAergic system. They increase the frequency of chloride channel opening after binding to GABA_A_ receptors, which eventually facilitates inhibitory signals. Zolpidem increases chloride influx after binding to BZ1 receptors near, but not at the GABA_A_ binding site of benzodiazepines in GABA_A_ receptor complexes.

#### Dopaminergic system

Medications primarily affecting the dopaminergic system can be divided into 1) levodopa and 2) dopamine reducing medications including presynaptic dopamine depletors (such as tetrabenazine [TBZ]) and postsynaptic dopamine blocking agents (DRBAs such as clozapine, quetiapine, and typical neuroleptics). The mechanism of action of levodopa in dystonia other than dopa-responsive dystonia (DRD) remains poorly understood. It appears counterintuitive that both levodopa and dopamine-reducing strategies provide benefit in dystonia.

TBZ inhibits the enzyme vesicular monoamine transporter 2 (VMAT2), thereby reducing transport of dopamine into presynaptic vesicles. Reserpine also inhibits VMAT2, but it has peripheral effects as well. Metyrosine (a.k.a. α-methyl-para-tyrosine or Demser®) inhibits tyrosine hydroxylase, a presynaptic enzyme required for dopamine synthesis.

DRBAs act by blocking dopamine receptors at postsynaptic sites. Typical neuroleptics generally have effects at D2 receptors, whereas atypical neuroleptics (e.g. clozapine and quetiapine) possess less risk of triggering acute dystonic reaction or tardive syndromes.

### The evaluation and initiation of medical treatment in dystonia

We present a practical approach for initiating medical treatment in a patient with dystonia (Table 1). We organize the discussion around four central questions.

**Table 1:** Practical guide for initiation of medications and selection of symptomatic medical therapies

*A. Questions to ask before initiating treatment:*	
1) “Does the patient really have dystonia?”	
	- Exclude pseudodystonia and psychogenic dystonia
2) “Is there any (etiology-) specific treatment for the patient?”	
	*-* Identify treatable dystonia (Table 2): neurometabolic disorders (DRD being the most important), heavy metal-related disorders (especially Wilson’s disease) and acquired disorders
3) Is there any coexisting phenomenology other than dystonia?	
	*-* Identify and appropriately treat coexisting phenomenology such as parkinsonism (e.g. in RDP) or myoclonus (e.g. in myoclonus-dystonia syndrome or DYT11 dystonia)
4) “What treatment modality or modalities should be initiated?”	
	- Selecting between medications vs. BoNT vs. DBS or combination (Table 3)
*B. General principles of symptomatic medical treatment in dystonia*	
	• Trihexyphenidyl is a first-line agent • Baclofen and clonazepam are typically second-line agent • TBZ or clozapine may be considered as first-line agents in tardive dystonia • Start low, go slow o Initiate at a low dose o Titrate up slowly ▪ Every 3–4 days in children and younger adults ▪ Every 1 week for older adults or patients prone to side effects • Continue uptitration if non-sustained benefits or inadequate symptom control • If side effects occur – may initially try holding the dose constant. If no improvement, or severe/intolerable side effects – lower the dose (modified from Ref [5]) o If side effects disappear and patient still benefits: ▪ Consider combination therapy or try increasing the dose slowly again o If side effects disappear but no benefit: ▪ Consider discontinuation (may need slow tapering especially baclofen and clonazepam) o If side effects persist but patient still benefits: ▪ Consider lowering the dose further o If side effects persist and no benefit ▪ Consider discontinuation (or slow tapering) o If benefits are seen and symptoms are adequately controlled: ▪ Hold constant to see if benefits are sustained. • Of note, sometimes trihexyphenidyl at a constant dose may require 2–4 weeks to reach peak benefit • Trihexyphenidyl may have paradoxical effects at low doses o If this occurs – may try pushing to higher doses slowly

A. Step-by-step approach before initiation of medical treatment in dystonia: a practical guide

B. General principles of symptomatic medical treatment in dystonia. Further detail is described in the review

*Abbreviations*: *BoNT*, botulinum toxin injection, *DBS* deep brain stimulation, *DRD* dopa-responsive dystonia, *RDP* rapid-onset dystonia parkinsonism, *TBZ* tetrabenazine

1. Does the patient really have dystonia?
  This is the first and most important question to answer before initiating treatment. Clinicians must be able to differentiate pseudodystonia and psychogenic dystonia from true dystonia. Useful clues for psychogenicity include rapid onset, fixed postures which do not vary over time, inconsistency and variability on exam. Some important examples of pseudodystonia include congenital torticollis (where surgical release of the fibrotic muscular tissue may be indicated), atlantoaxial subluxation (requiring urgent orthopedic management), and stiff limb syndrome (which requires immunotherapy).
2. Is there an etiology-specific treatment for the patient?
  This is the next step once the diagnosis of true dystonia is secured. Treatment in dystonia can be classified as etiology-based vs. symptomatic. While most treatments remain symptom-based, etiology-based treatments exist for a few forms of dystonia (“don’t-miss” diagnoses”) and can provide remarkable benefits. They can be grouped into three main categories: neurometabolic disorders, heavy metal-related disorders, and acquired dystonia (Table 2).

**Table 2:**
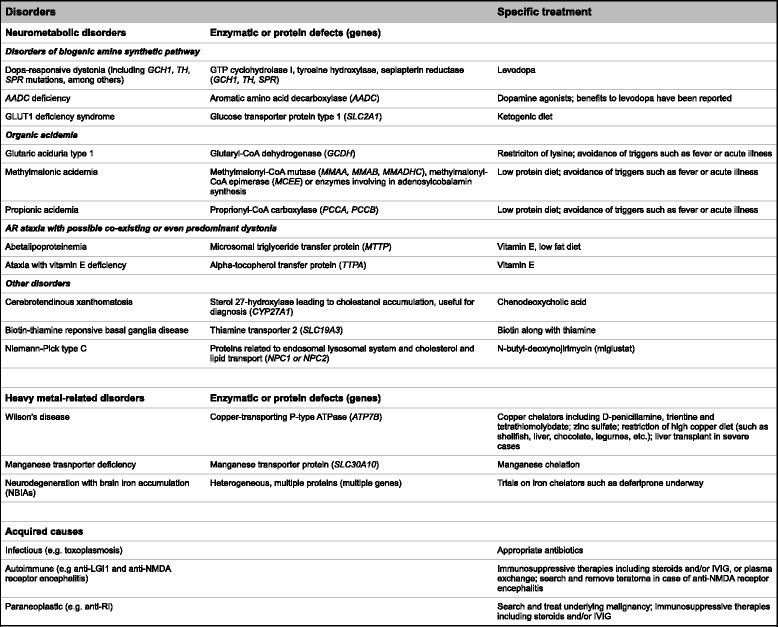
Dystonic disorders where etiology-specific treatment is available

Dystonic disorders where etiology-specific treatment is available

The disorders in this group can be categorized into neurometabolic disorders, heavy metal-related disorders and acquired disorders. The disorders in each subgroup are listed. The therapies are listed on the rightmost column. In the first two groups, the middle column demonstrated underlying enzymatic or protein defects with responsible genes in parentheses

*Abbreviations*: *AADC* aromatic amino acid decarboxylase, *GLUT1* glucose transporter type 1, *IVIG* intravenous immunoglobulin, *LGI1* leucine-rich glioma-inactivated 1, *NMDA* N-methyl-D-aspartate
  The quintessential “don’t-miss” diagnosis is DRD in which levodopa serves as an etiology-specific therapy. DRDs typically have dramatic and sustained response to levodopa [12], and their phenotypes are broad [13, 14]. DRD can present with focal, segmental or generalized dystonia in children, or with limb-onset focal or segmental dystonia in adults [13, 14]. The first responsible gene was discovered in 1994 when Ichinose first reported mutations in the *GCH1* gene encoding the enzyme GTP cyclohydrolase I [15]. This enzyme is essential in the synthesis of tetrahydrobiopterin (BH_4_), a cofactor required in the synthetic pathways of monoamines including dopamine and serotonin. Less common genes including *TH1* (encoding tyrosine hydroxylase, the rate limiting step in dopamine synthesis) and *SPR* (encoding sepiapterin reductase, another enzyme required for BH_4_ synthesis) were later discovered [16, 17].
  An observed levodopa trial (generally up to 300–400 mg of levodopa daily in adults or 4–5 mg/kg/day in children [18], for at least one month) is recommended in all children with any forms of dystonia, and adults whose phenotypes cannot exclude DRD. However, the dose ranges may vary depending on the genotypes e.g. as shown in one study, 100–400 mg/day in adult patients with *GCH1* mutations vs. 150–600 mg/day in adult non-*GCH1* patients [19]. Children with autosomal recessive forms of DRD such as autosomal-recessive *GCH1*, *TH* and *SPR* mutations may require higher dose (e.g. 6–10 mg/kg/day), as opposed to conventional dose, 4–5 mg/kg/day, in autosomal dominant *GCH1* mutations [14, 18]. Exposing patients to high doses (e.g. up to 1000 mg/day in adults or 16–20 mg/kg/day in children) [12, 14] is not usually recommended prior to genetic confirmation [18].
  DRD patients typically have an excellent and sustained response to levodopa [12, 20]. In the long term, patients usually stay on relatively low and stable (or even lower) doses in adulthood [12, 21]. Wearing off phenomenon and levodopa-induced dyskinesias are much less common than in Parkinson’s disease, but have been reported [12, 20, 22–24], particularly in autosomal recessive forms (e.g. *TH*, *SPR* mutations) as opposed to autosomal dominant DRD [14]. Levodopa-induced dyskinesias also tend to occur at higher doses, and are improved by dose reduction without worsening of motor functions. Additional therapies may be required in some forms of DRD such as 5-hydroxytryptophan (5-HTP, up to 6 mg/kg/day) in sepiapterin reductase deficiency [25, 26], and 5-HTP and BH_4_ in autosomal recessive *GCH1* mutations [27].
  Among heavy metal-related disorders, Wilson’s disease is the prototypical “don’t-miss” diagnosis. Treatment includes chelation therapies (D-penicillamine, trientine and tetrathiomolybdate) and zinc sulfate [28–31]. Among treatable neurometabolic disorders, cerebrotendinous xanthomatosis deserves special mention, and careful search for tendon xanthoma and blood levels of cholestanol are useful prior to genetic testing. It is treatable with chenodeoxycholic acid.
  Niemann-Pick type C can present with dystonia, in addition to ataxia and vertical supranuclear gaze palsy [32–34]. Treatment with miglustat (N-butyl-deoxynojirimycin) has been shown to improve or stabilize neurological manifestations [35, 36]. Neurodegeneration with brain iron accumulation (NBIA), another example of heavy metal-related disorders, has been reported to benefit from iron chelation with deferiprone [37, 38], although this needs further study.
  Even when an etiology-specific approach is available, symptomatic medical therapies can still be employed as an adjunct or bridging therapy until the specific treatment achieves maximal benefit. For example, in Wilson’s disease, anticholinergics can be used to symptomatically treat dystonia concurrently with copper chelation.
3. Is dystonia the only phenomenology? Or are there coexisting phenomenologies other than dystonia?
  “Dystonia-plus syndromes” [39, 40] or “combined dystonia” [41] have co-existing phenomenology such as parkinsonism and myoclonus. Identification of associated phenomenologies may have important implications for treatment. For example, dystonia associated with parkinsonism can be found in DYT3 dystonia (Lubag disease), DTY12 dystonia (rapid-onset dystonia parkinsonism, RDP), and NBIA.
  In DYT11 dystonia (myoclonus-dystonia syndrome), myoclonus may predominate, and symptomatic control is sometimes achieved by treating the myoclonus. Data is limited by a small number of reported patients and limited number of controlled trials. Given the subcortical origin of the myoclonus, it is reasonable to use clonazepam [42–46] or levetiracetam [46]. Data in double-blind placebo-controlled trials is unavailable, and in our experience levetiracetam has benefitted some patients (unpublished data). A recent randomized controlled trial in 23 patients demonstrated improvement of myoclonus with zonisamide [47]. Other medications reported in small studies include sodium oxybate [48, 49], tetrabenazine [50], anticholinergics (which improved only dystonia but not myoclonic component) [43], among others. Valproic acid was found to be ineffective in several studies [43, 51]. In severe medically refractory cases, pallidal (GPi) deep brain stimulation (DBS) should be considered [52–56], earlier rather than later [57, 58].
4. What treatment modality or modalities should be initiated?
  As a general rule, less invasive modalities such as medications and/or BoNT are usually tried before DBS, although the dramatic response of DYT1 generalized dystonia or DYT11 dystonia to DBS supports early intervention [52–61]. The list of indications for DBS in dystonia has been expanding. Some examples are DYT3 dystonia [62–66], cerebral palsy [67, 68], pantothenate kinase-associated neurodegeneration [69, 70] and idiopathic cervical dystonia [71].
  The decision whether to use oral medication vs. BoNT depends on the distribution of dystonia. For example, BoNT is first-line therapy in cervical dystonia, blepharospasm or spasmodic dysphonia, due to its excellent efficacy and tolerability. BoNT is usually employed first in focal or segmental dystonia where a limited number of muscles can be targeted. In generalized dystonia, BoNT may be of use in focal areas in order to relieve discomfort and improve function, such as injecting the hands in dystonic cerebral palsy. However, oral medications are almost always required.
  We summarize the treatment modalities for each form of dystonia in Table 3. Given the relative rarity and heterogeneity of dystonia, there have been only a handful of double blind randomized placebo controlled (DBPC) studies, and much of the evidence supporting these recommendations is level 4. Thus it is important to maintain flexibility in individualizing treatment.

**Table 3:**
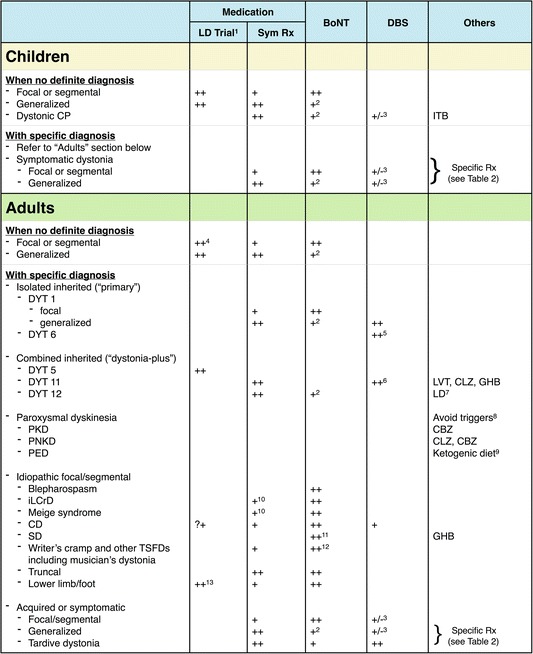
Summary of selection of treatment modalities in dystonia

After excluding disorders with etiology-specific therapies as shown in Table 1, symptomatic therapy in this table is then considered. This table summarizes treatment modalities in each dystonic disorder. Three major treatment modalities include medications, botulinum toxin injections and deep brain stimulation, among others. Of note, since DRD is an important “don’t-miss” diagnosis, “levodopa trial” is also included here under “Medication”. Of note, levodopa in this case serves for diagnostic and therapeutic purposes (“etiology-specific therapy”). In DYT5 or DRD, levodopa is the specific therapy which typically leads to dramatic and sustained benefit. If levodopa is used for other purposes such as “symptomatic therapy” for dystonia or coexisting parkinsonism, it is shown under “Others”

“++” represents first-line modality or, for DBS, there is a low threshold to consider; “+” represents second-line or adjunctive modality when used as a combination therapy; “+/−” means benefits remain unclear. “?+” means that levodopa trial in cervical dystonia is questionable: it may be considered but not as strongly indicated as in limb-onset or generalized dystonia in adults

^1^LD trial in this case includes only for diagnostic and therapeutic purposes, particularly when DRD is suspected or cannot be excluded. LD as a symptomatic therapy is shown under “Others”

^2^BoNT can be used to target focally at the most debilitating muscle group(s) e.g. to relieve discomfort or improve range of motion for rehabilitation or hygiene

^3^DBS has been reported. These disorders are expanding indications for DBS, and may require further studies to confirm benefits

^4^Especially the ones with limb-onset dystonia

^5^DBS in DYT6 dystonia is generally less beneficial than DYT1 dystonia, however it has still been performed

^6^Pallidal (GPi) stimulation relieves both dystonia and myoclonus

^7^LD in this case is used as a symptomatic therapy to treat coexisting parkinsonism

^8^Avoding triggers is helpful in paroxysmal dyskinesias such as avoiding caffeine, alcohol and sleep deprivation in PNKD, and avoiding strenuous exercise in PED

^9^Need to search for GLUT1 deficiency syndrome in PED. Ketogenic diet can be initiated once the diagnosis is confirmed

^10^Especially when there is involvement of complex muscle groups or tongue

^11^Adductor spasmodic dysphonia (ADSD) typically has better response to BoNT than abductor spasmodic dysphonia (ABSD)

^12^Not recommended in embouchure dystonia

^13^In order to rule out DRD, and also Parkinson’s disease presenting with foot or lower limb dystonia

*Abbreviations*: *BoNT* botulinum toxin injection, *CBZ* carbamazepine, *CD* cervical dystonia, *CLZ* clonazepam, *CP* cerebral palsy, *DBS* deep brain stimulation, *GHB* γ-hydroxybutyric acid or sodium oxybate, *GLUT1* glucose transporter type 1, *GPi* globus pallidus interna, *iLCrD* idiopathic lower cranial dystonia, *ITB* intrathecal baclofen, *LD* levodopa, *LVT* levetiracetam, *PED* paroxysmal exercise-induced dystonia, *PKD* paroxysmal kinesigenic dyskinesia, *PNKD* paroxysmal non-kinesigenic dyskinesia, *Rx*, treatment, *SD*, spasmodic dysphonia, *Sym Rx* symptomatic treatment, *TSFD* task-specific focal dystonia

### Medication selection and treatment strategy

#### General considerations

The strategy, developed by Fahn [5], is to “start low and go slow”: medications should be started at a low dose, and titrated up slowly to the lowest dose that is effective for sufficient symptom control without side effects. The rate of titration may depend on age: every 3–4 days in children, compared to every 1 week in adults. If symptoms are still not adequately controlled or benefits are not sustained, medications can be titrated up further. Should side effects emerge, we may try holding the dose constant until they disappear, but oftentimes reduction of the dose is needed. If side effects are severe, intolerable or persist, the medications should be lowered.

A combination approach is used when monotherapy achieves a “good” dose but symptom control is incomplete, or dosage is impeded by side effects. Anticholinergics may sometimes have a paradoxical effect i.e. worsening of dystonia at low dose which will disappear at high dosage [5], possibly due to pre-synaptic inhibition. In this circumstance, pushing the dose higher slowly may be considered, with close monitoring for side effects. Peak effect or benefits of some medications such as trihexyphenidyl may not be evident until the dose is held constant for at least 2–4 weeks [72].

**Fig. 2:**
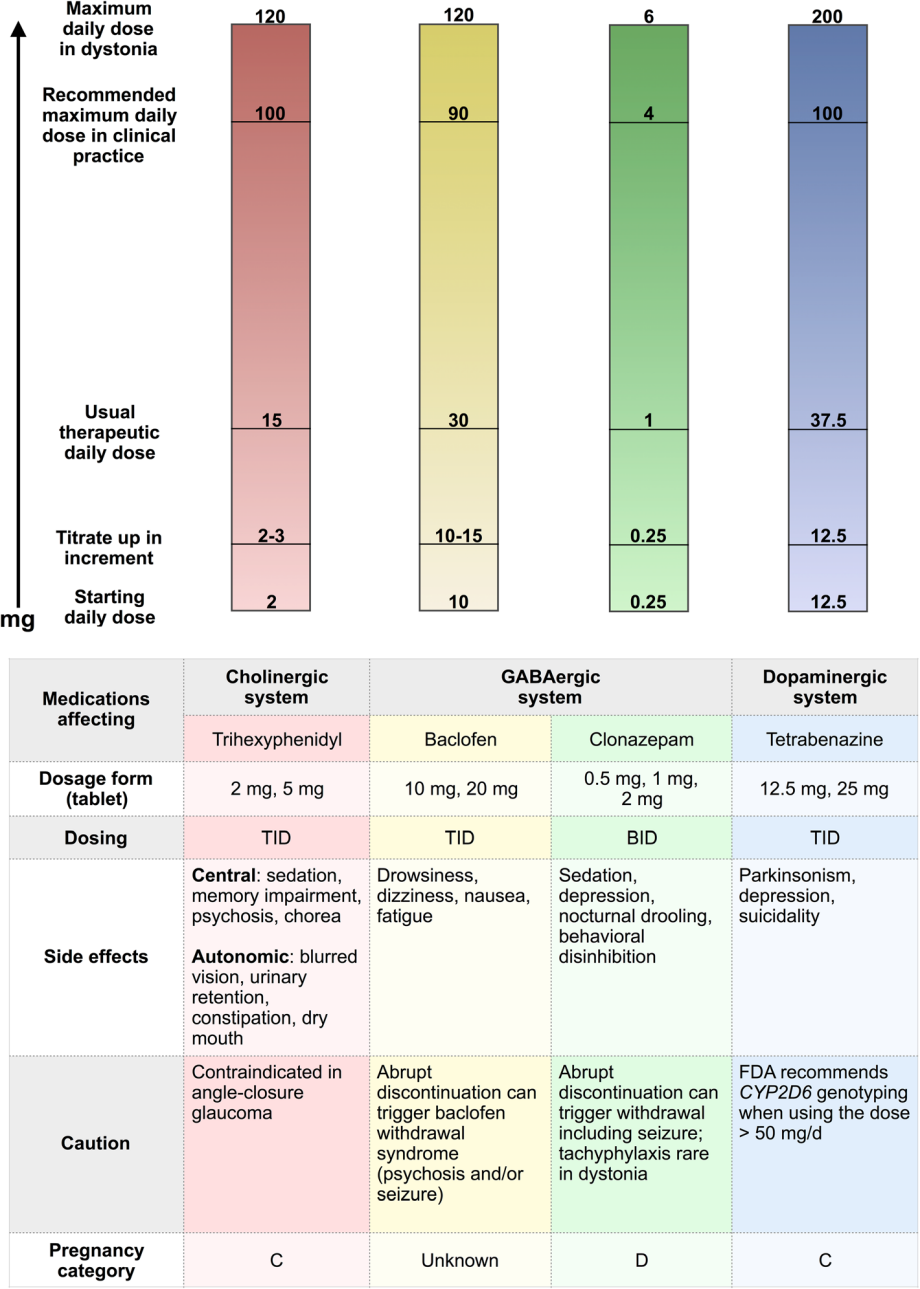
Diagram of major medications for symptomatic therapy in dystonia classified by their neurotransmitter systems. The corresponding bars show starting doses, titration doses (amount to increase with each titration period), usual therapeutic doses, our recommended maximum doses in clincial practice and ceiling doses. Of note, the bars do not represent the acutal scales. The table summarizes dosage forms, dosing, side effects, caution and Food and Drug Administration (FDA) pregnancy category of each medication. Levodopa is not included here as its major role in dystonia is for etiology-specific therapy as mentioned in Table 2 and the text. Abbreviations: mg, milligram; TID, three times a day; BID, two times a day

The dose ranges and titration schedule of the major medications are summarized in Fig. 2. In the 1980s, anticholinergics were used at high dosage [5, 6, 72]. However, in our current clinical practice, it is not very common to have patients on trihexyphenidyl higher than 30–40 mg daily, compared to up to 120 mg/day in the trials. Use of anticholinergics at high dosage is limited by side effects. For levodopa use in non-DRD dystonia, the dose and titration are similar to their use in mild Parkinson’s disease.

Which medications should be started first? Most clinicians in the U.S. use trihexyphenidyl as their first-line agent (Table 1B). However there are no available head-to-head comparisons with other agents in randomized placebo controlled trials. Baclofen may have a more important role in childhood dystonia with associated spasticity, such as in cerebral palsy [73]. Nonetheless, we suggest initiation with trihexyphenidyl if control of dystonia is a priority. If side effects occur or clinical benefits are not achieved, switching to or adding baclofen may be entertained. Clonazepam is generally considered a second- or third-line agent.

Of note, paroxysmal dyskinesias are unique, and the therapeutic approach is quite different from other dystonias: antiepileptics and avoiding triggers are the mainstay therapy. The first important step is to exclude secondary paroxysmal dyskinesias (such as from demyelinating diseases, parathyroid disorders and vascular lesions including Moyamoya syndrome) [74–78] especially in atypical age group or adults, since specific treatment of underlying disorders are indicated. Three main types of primary paroxysmal dyskinesias include paroxysmal kinesigenic dyskinesia (PKD), paroxysmal nonkinesigenic dyskinesia (PNKD) and paroxysmal exercise-induced dyskinesia (PED). The most common mutations in PKD and PNKD are in the *PRRT2* [79] and *PNKD* (a.k.a. *MR-1*) [80, 81] genes, respectively. Some PED patients carry mutations in the *GLUT1* or *SLC2A1* gene [82], and are considered to be in the phenotypic spectrum of glucose transporter type 1 deficiency syndrome (Glut1 DS).

PKD patients often respond completely to low dose carbamazepine (50–400 mg/day in adults or 1.5-15 mg/kg/day in children) [83–85], or less commonly used phenytoin (150–300 mg/day in adults or 5 mg/kg/day in children) [85–87]. The dosage for PKD is typically lower than that in epilepsy. FDA recommends the HLA-B*1502 in patients at high risk of Steven-Johnson syndrome or toxic epidermal necrolysis such as those of Asian descent prior to initiation of carbamazepine [88]. Other antiepileptics such as oxcarbazepine [83, 89–92], levetiracetam [93], gabapentin [94], topiramate [95], valproic acid [96] and lamotrigine [97–99] have been reported. Acetazolamide has been reported in secondary PKD due to demyelinating diseases [100]. PNKD patients should avoid precipitating factors such as caffeine, alcohol and sleep deprivation. The response to anticonvulsants is less favorable in PNKD compared to PKD [101, 102]. Clonazepam (2–4 mg/day) may be used as first-line treatment [101, 103–105]. Other medications such as acetazolamide [105], haloperidol [106], gabapentin [104] and oxcarbazepine [107] have been reported in PNKD. PED patients should avoid prolonged exercise, and PED patients with Glut1 DS can benefit from the ketogenic diet [82, 108]. Management of dystonic storm will not be discussed here.

The history of the medical treatment of dystonia offers many valuable lessons. Serendipity has played an important role in the development of medical treatment. In the following section, we will start the discussion of medications affecting each neurotransmitter with a historical review.

#### Medications primarily affecting the cholinergic system

In 1952, beneficial effects of trihexyphenidyl in writer’s cramp and “dystonia musculorum deformans” were first reported [109, 110]. In 1983, Fahn conducted the first open-label study of high dose anticholinergics in dystonia using trihexyphenidyl and ethopropazine [5]. Anticholinergics were employed in various forms of dystonia, both “primary and secondary”, excluding tardive dystonia and Meige syndrome. Benefits were found in 61% of the children and 38% of the adults, with average trihexyphenidyl doses of 41 and 24 mg respectively. In 1986, a prospective DBPC crossover trial of anticholinergics in children and young adults employed doses up to 120 mg/day of trihexyphenidyl [72]. A clinically significant response was seen in 71% of a total of 31 patients, and benefit remained in 42% of the patients after 2.4 years of follow up. More benefit was demonstrated in children, possibly due to better tolerability, and in patients who received treatment earlier, within 5 years of disease onset [5, 6, 72].

Anticholinergics have been shown to benefit various forms of dystonia including focal [111], cranial dystonia [112], and secondary dystonia including dystonia in cerebral palsy [72, 113–115], after ischemic stroke [116] and in tardive dystonia [114, 117].

The adverse effects of anticholinergics encompass central and autonomic symptoms (Fig. 2). Central side effects include sedation, cognitive slowing, confusion, memory impairment, psychosis and chorea. Autonomic side effects include blurred vision due to mydriasis, dry mouth, urinary retention and constipation. Anticholinergics are contraindicated in patients with a history of acute angle-closure glaucoma. Certain autonomic side effects may be relieved by using pilocarpine eye drop (for blurred vision), or pyridostigmine for other symptoms. Abrupt discontinuation of anticholinergics has been reported to rarely worsen dystonia and can even trigger life-threatening cholinergic crisis [118]. In clinical practice, the issue of anticholinergic withdrawal does not appear to be as concerning compared to baclofen or benzodiazepines. Nonetheless, we recommend tapering anticholinergics very slowly.

#### Medications primarily affecting the GABAergic system

There have been no DBPC trials of baclofen or benzodiazepine in dystonia. Baclofen was used in spasticity before it was applied to dystonia. In 1982, Brennan reported improvement of Meige syndrome with a combination of sodium valproate and baclofen [119]. Afterwards baclofen was reported to be useful in tardive dystonia [120]. Greene published a retrospective open label trial in 1988 [6]. Twenty percent of 108 patients had benefits from baclofen. The dose ranged from 25 to 120 mg/day (mean 82 mg/day). There was a trend of more benefit in blepharospasm compared to other forms of dystonia. Later, Greene and Fahn also reported beneficial effects of baclofen in seven of 16 patients with idiopathic childhood dystonia [121]. Some who responded to baclofen did not have a good or sustained response to anticholinergics. In line with the previous study [6], a better response to baclofen correlated with shorter duration of therapy, especially when initiated within 3 years of symptom onset.

Due to limited cerebrospinal fluid penetration [122], intrathecal baclofen (ITB) was tried, initially for spasticity [123, 124], and later in dystonia [125–131]. Efficacy of ITB was initially reported in intractable axial dystonia by Narayan in 1991 [129], and subsequently in dystonic cerebral palsy with lower extremity involvement [130, 131]. The benefits to the lower limbs [132] may be associated with the gravity-induced concentration gradient in the thecal sac [133]. Albright reported intraventricular baclofen (IVB) use in two patients with dystonic cerebral palsy, one of whom previously failed ITB therapy and the other with a complex spinal anatomy precluding the intrathecal procedure [134]. Benefits of IVB in other secondary dystonias such as glutaric aciduria type 1 have been reported [135]. IVB is a useful alternative in patients with refractory dystonia requiring multiple revisions of ITB pump [136]. Before pump placement, patients generally receive a trial dose of ITB in order to assess their expected response. ITB has become less popular in dystonia due to the complication rates from the surgical procedures and hardware-related problems including infection or pump malfunction. In addition, abrupt discontinuation of ITB can lead to dystonic storm or baclofen withdrawal syndrome, which may be life-threatening.

Baclofen is generally considered as a second-line agent. Side effects include drowsiness, dizziness, fatigue and nausea. Its use in dystonia with coexisting spasticity may lead to excessively reduced muscle tone. It is critical to gradually taper the dose down and not to abruptly discontinue ITB in order to prevent the baclofen withdrawal syndrome.

With regard to benzodiazepines, diazepam therapy was reported in “dystonia musculorum deformans progressiva” and spasmodic torticollis [137–139]. In 1988, Greene found benefit of clonazepam in 16% of 115 patients with various forms of dystonia, including secondary dystonia [6]. In one study of 33 patients with acquired hemidystonia, clonazepam and diazepam were found to be the most effective drugs, although others reported more benefit with anticholinergics [140]. No head-to-head comparisons between various benzodiazepines are available.

In general, benzodiazepines are considered to be a second- or third-line agent. Clonazepam and diazepam are the two most commonly used drugs, partly due to their relatively long half-lives. Side effects of benzodiazepines include sedation, depression, nocturnal drooling and behavioral disinhibition. As with baclofen, abrupt discontinuation can lead to a withdrawal syndrome and seizures. Tachyphylaxis has been reported in other indications [141], although to our knowledge not in dystonia.

#### Medications primarily affecting the dopaminergic system

Levodopa therapy entered the field of dystonia in 1976 when Segawa first reported a dramatic response to low dose levodopa in two female cousins with “hereditary progressive dystonia with marked diurnal fluctuations” [21], subsequently named Segawa syndrome or DRD. Indeed, Beck originally described in 1957 an 8.5-year-old girl with “dystonia musculorum deformans” [142] who was noted to have diurnal fluctuations later by Corner [110]. The gene discoveries were already discussed above.

Levodopa can serve two different purposes in dystonia therapy: 1) as an etiology-specific treatment in DRD, and 2) as a symptomatic therapy in other forms of dystonia where the dramatic response to levodopa is unfortunately not replicated. It may also be used to treat parkinsonism that coexists with dystonia such as in RDP [143]. In our practice, we rarely use levodopa or dopamine agonists to treat dystonia symptomatically. Side effects of levodopa include nausea, orthostatic hypotension and psychosis. However in DRD it is typically administered at low doses, so side effects are less frequent. Levodopa-induced dyskinesias are uncommon in DRD.

In 1972, Swash reported only slight benefit of TBZ in spasmodic torticollis [144]. In 1982, a double blind crossover trial by Jankovic demonstrated improvement in 11 of 12 patients [145]. TBZ has been used in various forms of dystonia and other hyperkinetic movement disorders [145–149], however benefits are greater in tardive dystonia compared to other forms [147]. TBZ was not available in the U.S. until FDA approval in 2008 for chorea associated with Huntington’s disease, and it has been used off-label in a variety of hyperkinetic movement disorders including dystonia [150].

TBZ is rarely used as a first-line agent, except in tardive dystonia [150]. Reserpine is rarely used for dystonia due to its peripheral side effects (e.g. hypotension). Clinical use of metyrosine [151] is restricted by its availability. The most important adverse effects of TBZ are parkinsonism and depression. The FDA label recommends *CYP2D6* genotyping when using TBZ above 50 mg/day [152]. Patients with exceptionally high activity of CYP2D6 enzyme, called ultrarapid metabolizers, can theoretically have short pharmacological effects. One study found that ultrarapid metabolizers needed longer titration and tended to require higher dosages [153].

Deutetrabenazine (SD-809) is a new medication that is structurally related to TBZ and thus has similar action as a VMAT2 inhibitor. The added deuterium molecule attenuates CYP2D6 activity, thereby prolonging its half-life. A trial is underway for chorea in Huntington’s disease [154], and it is likely that the indications may expand to tardive dyskinesias and dystonia in the future.

While DRBAs can cause acute dystonic reactions and tardive dystonia, the paradox is that they can sometimes improve dystonia. Conflicting data revealed benefits in some studies, particularly in tardive dystonia [146, 155–157], but not in others [158, 159]. The use of the DRBAs as routine treatment for dystonia is generally discouraged due to the risk of engendering tardive syndromes [7, 160–162]. DRBAs acting at D2 receptors carry higher risks compared to the ones acting at others, including D4. Clozapine is considered to be a “true” atypical neuroleptic: it blocks the D4 receptor without blocking D2, and tardive syndromes have not been reported with clozapine. Quetiapine has been rarely reported to cause tardive dyskinesia, nevertheless in clinical practice it is used more frequently since clozapine requires frequent blood draws to monitor for agranulocystosis.

In our clinical practice, we generally preserve dopamine reducing agents for tardive syndromes or for patients with generalized dystonia who have not obtained adequate benefit from other medications. We choose TBZ first. If it is ineffective or intolerable side effects ensue, our next preferred treatment is clozapine.

#### Other agents

A variety of medications from small trials or case reports include other muscle relaxants such as carisoprodol or tizanidine, anticonvulsants such as sodium valproate, carbamazepine and phenytoin, as well as L-tryptophan and riluzole. Some medications, such as mexilitine and 5-hydroxytryptophan, have largely disappeared from current clinical practice. We will discuss three selected medications which have been reported to have large clinical benefits in small numbers of patients: sodium oxybate (or sodium γ-hydroxybutyrate [GHB]; Xyrem®), zolpidem, and cannabinoids.

Sodium oxybate, a GABA derivative, acts as a GABA_B_ and GHB receptor agonist. It was approved in the U.S. for treatment of cataplexy in narcolepsy in 2002. Its biological effects similar to ethanol render applications in several alcohol-responsive movement disorders including essential tremor, myoclonus-dystonia syndrome, spasmodic dysphonia (SD), as well as posthypoxic myoclonus [48, 49, 163–165]. Frucht reported beneficial effects treating three patients with myoclonus-dystonia syndrome who were alcohol responsive [49]. However alcohol abuse and dependence are not uncommon in DYT11 patients [166], and the drug may not be safe in this population. Alcohol responsiveness has been reported in about 55-60% of patients with SD [167]. In one case report [164] as well as ongoing experience in our center (unpublished data), sodium oxybate has beneficial effects in SD patients who are alcohol-responsive. Interestingly, one patient had a prolonged effect for several months after a single dose of sodium oxybate [164]. Off-label use of sodium oxybate in clinical practice may be challenging due to limitation in insurance coverage and strict substance control.

Zolpidem was initially claimed as a non-addicitive non-benzodiazepine hypnotic drug. Unfortunately, addictive potential has been reported with zolpidem [168]. The very first reports of zolpidem use in movement disorders were in patients with Parkinson’s disease and progressive supranuclear palsy [169, 170]. Evidente reported benefits in three patients with DTY3 dystonia [171]. Later reports of zolpidem in different forms of dystonia included focal, segmental and generalized dystonia [172–176]. Miyazaki found 28, 18 and 31% improvement in generalized, Meige syndrome/blepharospasm and hand dystonia, respectively [172]. The dose ranged from 5 to 20 mg/day with an average dose of 8–12 mg/day. The main side effect that limits dose is sedation.

Medical cannabinoids have become a popular topic since some states in the U.S. have legally approved cannabis use [177, 178]. Cannabinoids refer to a group of medications that act at the cannabinoid receptors, CB1 and/or CB2. CB1 receptors are primarily present in the brain, particularly in basal ganglia, limbic system, cerebellum and cerebral cortex. CB2 are mainly found in the spleen, tonsils, bone marrow and peripheral leukocytes. CB1 receptors are located at the presynaptic glutamatergic and GABAergic terminal axons innervating the striatal MSNs, as well as at the MSNs’ terminal axons [179, 180]. Activation of CB1 receptor leads to gluatamate release.

Cannabinoids can be derived from plants (phytocannabinoids e.g. delta-9-tetrahydrocannobinoid [THC], cannabinol and cannabidiol), synthesized (synthetic cannabinoids e.g. nabilone) or produced within the human body (endocannabinoids). Dronabinol (Marinol®) is a synthetic THC. THC has psychoactive effects such as euphoria, whereas cannabidiol has more sedative antiemetic and analgesic effects.

Several studies showed mixed results of cannabinoids in various forms of dystonia [181, 182]. Some showed benefit [183–185], whereas two small randomized DBPC trials did not [186, 187]. Benefits of cannabinoids in dystonia were concluded as “unknown efficacy”, according to an American Academy of Neurology (AAN) systematic review [178]. Anecdotally, benefit of THC has also demonstrated in one case of musicians’ hand dystonia (Altenmüller, personal communication). Further clinical experience and studies are required.

### The evolving role of medical treatment in dystonia: uncertainty for clinicians

With advances in diagnosis and treatment, therapeutic strategies including pharmacological management in dystonia have evolved. Progresses in other areas such as BoNT, neuromodulation and disease-specific treatment have changed the way patients are treated. Medical treatment in dystonia now is quite different from two decades ago. The following practical questions may help guide the clinician embarking on medical treatment of dystonia.

**Q:** *Should patients with all forms of dystonia be treated the same way?*

**A:** No. Etiology-specific approach should be contemplated first if there are known treatable etiologies (Table 2). In DRD, levodopa use is diagnostic and may produce dramatic response. In combined dystonia, co-existing phenomenology such as parkinsonism in DYT12 dystonia or myoclonus in DYT11 dystonia should be taken into consideration. In idiopathic isolated or primary dystonia where etiology-specific approaches are unavailable, symptomatic treatment is the norm. A suggested therapeutic scheme appears in Table 3.

**Q:** *Does the distribution of dystonia affect choice of therapy and response?*

**A:** BoNT is preferred in focal or segmental dystonia, whereas medical therapy is a first choice for generalized dystonia. In focal/segmental dystonia, medical treatment may have a primary role when BoNT is not technically feasible, or where BoNT is prone to side effects or functional impairment such as tongue or embouchure dystonia [188].In general, location of dystonia does not impact the choice of medication. As discussed, we start with an anticholinergic as a first-line, and baclofen or clonazepam as a second-line agent. Some experts may prefer one medication to another in some forms of dystonia based on clinical experience, for example benzodiazepines in hemidystonia [140].

**Q:** *Do adults and children respond the same way to treatment?*

**A:** Probably not, although evidence is lacking. Children tolerate higher doses of medications than adults.

**Q:** *Should every patient have a witnessed trial of levodopa?*

**A:** Not necessarily. The rule of thumb is if DRD cannot be ruled out, a levodopa trial is essential. Generally, we recommend a levodopa trial in all children or adults with generalized or focal limb dystonia, since this could represent an atypical phenotype of DRD. If patients already have a clear alternative diagnosis, a levodopa trial is not necessary.

**Q:** *How should medications be used in the modern era of BoNT? During what period: prior, concurrent or when BoNT wears off?*

**A:** As seen in Table 3, in some forms of focal dystonia such as blepharospasm, SD or cervical dystonia, BoNT is typically used first.

**Q:** *How should medications be used in the modern era of surgery?*

**A:** Typically almost all patients undergo medication trials at some point prior to the decision to undergo DBS surgery. There is a modern trend to pursue surgery earlier in the disease course. This is particularly applicable to disorders such as DYT1 generalized dystonia or DYT11 dystonia. After DBS surgery, medications are usually left unchanged until the initial programming. On the day of the initial programming, we ask the patient to hold one or two doses of the medication(s). Once benefits from DBS are seen (which may take months in DYT1 dystonia), we then taper down the medication(s) slowly.

## Conclusion

Dystonia remains a challenging field in both diagnostic and therapeutic aspects. Further understanding of its pathophysiology may shed light on more specific therapies. Symptomatic medical therapy can improve quality of life and should not be overlooked.

Video Legends:

- Video segment 1: Video segment 1 demonstrates examples of dystonia mimics and psychogenic dystonia. *Patient 1* was referred for evaluation of head tilt, concerning for “cervical dystonia”. However, on examination the left head tilt was relatively fixed even when she turned her head in different directions. Palpation revealed a harder-than-normal consistency of her left sternocleidomastoid muscle due to fibrosis. She was referrred for surgical evaluation after our diagnosis of congenital muscular torticollis. *Patient 2* had a history of previous trauma to the left foot several months prior. Examination demonstrated relatively fixed inversion of the left foot when sitting and walking, and no improvement when walking backwards. She was diagnosed with complex regional pain syndrome. *Patient 3* presented with difficulty moving the left leg. Examination revealed a slight increase in tone of the left leg to passive range of motion, as well as a slightly brisker quadriceps reflex on the left. His left leg was slightly more clumsy than the right with active range of motion and when walking. The diagnosis of stiff limb syndrome, a variant of stiff person syndrome, was confirmed by marked elevation (>250 U/ml) of anti-glutamic acid decarboxylase (anti-GAD) antibody. The next videos demonstrate a variety of psychogenic dystonias including psychogenic jaw dystonia presenting with left jaw deviation (*Patient 4*), psychogenic laryngeal dystonia with choking-like symptom (*Patient 5*), psychogenic upper and lower limb dystonia (*Patient 6*), psychogenic left foot dystonia (*Patient 7*), and psychogenic right hand dystonia (*Patient 8*).
- Video segment 2: Video segment 2 demonstrates select examples of “don’t-miss” diagnoses. *Patient 1* is a teenage girl with Wilson’s disease. She had prominent lower facial dystonia, a risus sardonicus, and a jerky 6-Hz tremor of the left fingers. *Patient 2* also has a diagnosis of Wilson’s disease. She presented with cervical dystonia, left shift, slight head tilting to the right (with her chin pointing to the left) and prominent right shoulder elevation. Lateral rotation of her neck to the right was more difficult than to the left. A low amplitude dystonic jerky head tremor was intermittently seen. Her parkinsonian features including facial masking and bradykinesia on finger tapping were mild. A classic Kayser-Fleischer ring was demonstrated as a relatively thick brown rim along the entire circumference of the limbus. Both Patients 1 and 2 experienced marked benefit from oral copper chelating therapy. *Patient 3* is a 10-year-old girl with a genetically confirmed mutation in the *ATP1A3* gene. She presented with an intermediate phenotype between alternating hemiplegia of childhood and rapid-onset dystonia parkinsonism [143]. The home video demonstrated an episode of upward oculogyria with slight head tilting to the left, during which her consciousness remained intact. Oculogyric episodes disappeared when she was treated with levodopa 300 mg/day, but moderate residual dystonic posturing of the left hand was still present.
- Video segment 3: Video segment 3 demonstrates example of patients with primary dystonia. The first three patients have childhood-onset genetically confirmed DYT1 dystonia with predominant foot or leg involvement. *Patient 1* was unable to stand or walk independently due to bilateral foot dystonia. With 15 mg/day of trihexyphenidyl and 30 mg/day of baclofen, she was able to stand and take a few steps with minimal support. *Patient 2* demonstrated flexion dystonia of her left more than right foot that was moderately improved with trihexyphenidyl 45 mg/day and baclofen 30 mg/day. *Patient 3* demonstrated prominent inversion of the left foot, with much milder right foot dystonia. His dystonia was more prominent with activation such as during the swing phase of walking. With trihexyphenidyl 10 mg/day, the left foot dystonia improved only slighlty, and the residual right foot dystonia was minimal. *Patient 4* is a girl who presented with dystonia, negative for *DYT1* and *DYT6* mutations. Her dystonia involved the trunk and, to a lesser degree, all extremities. Prominent truncal twisting was demonstrated even when she was sitting in the chair. This improved with trihexyphenidyl 30 mg/day, but she still had residual truncal dystonia seen as pelvic tilting when walking. She ultimately underwent bilateral GPi DBS surgery with marked improvement in her dystonia.
- Video segment 4: Video segment 4 demonstrates dopa-responsive dystonia (DRD; *Patients 1–2*), one of the most crucial “don’t-miss” diagnoses in dystonia, and paroxysmal kinesigenic dyskinesia (*Patients 3–5*). These two disorders are treated with, in respective order, low dose levodopa and low dose carbamazepine with dramatic benefits. *Patient 1* had residual minimal dystonia of her feet after she was given a low dose levodopa. *Patient 2*, her mother, unfortunately underwent the orthopedic surgical corrections of her deformities in childhood due to a missed diagnosis. Recognition of DRD and a levodopa trial would have prevented her from these unnecessary invasive procedure. *Patient 3* had an episode of paroxysmal dystonia involving mainly his trunk (extension and flexion) lasting a few seconds while standing. The second home video displayed another episode of dystonia involving both hands and arms while he was sitting. He returned to his normal state after each episode. *Patient 4* presented with episodes of truncal dystonia. The one captured in this home video demonstrated truncal twisting while standing. He held the chair during the episode which lasted several seconds before returning to his normal baseline. Home videos of *Patient 5* demonstrate episodes of dystonia primarily involving both legs and feet. Each episode lasted a few seconds. The first one occurred while he was sitting in the bed. During the second episode, he deliberately made a step with his right foot due to paroxysmal dystonia.
- Video segment 5: Video segment 5 demonstrates a variety of focal dystonias. The first two patients have velopharyngeal dystonia, a form of focal task-specific dystonia involving pharyngeal muscles and soft palate (velopharynx) which leads to inabilty to close the air passage connecting the mouth and nasal cavity, producing a nasal quality of speech. *Patient 1* is a 41-year-old woman who presented with breathy and hypernasal voice. Stroboscopic examination of her larynx did not reveal evidence of spasmodic dysphonia. She was started on trihexyphenidyl which was titrated up to 12 mg/day, and on follow-up visit two months later her speech improved by approximately 75%. *Patient 2* is a 61-year-old man who presented with two years of nasal speech. Marked improvement with trihexyphenidyl 12 mg/day was seen. *Patients 3 and 4* demonstrate a variety of idiopathic lower cranial dystonias (iLCrD). Further details about this particular topic can be found in Ref [189]. *Patient 3* demonstrates pure left jaw deviation aggravated by speaking, improved with trihexyphenidyl 2 mg/day. Patient 4 is a woman with severe jaw opening dystonia triggered by speaking. Light touching of her chin served as a geste antagoniste that improved her dystonia. She tried multiple medications including trihexyphenidyl, baclofen, clonazepam and leveteiracetam without success due to side effects before achieving an adequate response with diazepam 15 mg/day.
- Video segment 6: Video segment 6 demonstrates a woman with left hemidystonia. She placed her left hand underneatth her left leg in order to compensate. When walking, dystonia of the left arm, seen as flexion of the left elbow, extension of the left wrist and flexion of her fingers was demonstrated. The left arm braced her trunk while walking with marked reduction in arm swing. On follow up visit several years later, on lorazepam 2 mg/day, she had moderate dystonic posturing of the left hand at rest and when holding her arms in outstretched position. Mild dystonic posturing of the left foot was also demonstrated when sitting. Dystonic posturing of the left arm was less prominent compared to the previous examination.
- Video segment 7: Video segment 7 demonstrates a variety of tardive dystonias. Both patients had a history of dopamine receptor blocking agent use in the past. *Patient 1* had phasic cervical dystonia, characterized by intermittent jerky anterior neck shifting in commbination with neck flexion. Dystonia of the forehead muscles included frontalis and procerus involvement. The dystonia was alleviated by lightly touching her face with her hand. *Patient 2* demonstrated intermittent truncal flexion when sitting and walking. He underwent bilateral pallidal DBS surgery with resolution of his truncal dystonia.

## Abbreviations

5-HTP: 5-hydroxytryptophan
AAN: American Academy of Neurology
ACh: Acetylcholine
BH_4_: Tetrahydrobiopterin
BoNT: Botulinum toxin injections
ChI: Cholinergic interneuron
DBPC: Double blind randomized placebo controlled
DBS: Deep brain stimulation
DRBA: Dopamine receptor blocking agent
DRD: Dopa-responsive dystonia
FDA: Food and Drug Administration
GABA: γ-aminobutyric acid
GHB: γ-hydroxybutyrate
Glut1 DS: Glucose transporter type 1 deficiency syndrome
GPi: Globus pallidus interna
ITB: Intrathecal baclofen
IVB: Intraventricular baclofen
mg: milligram(s)
mg/kg/day: milligram(s)/kilogram/day
MSN: Medium spiny neuron
NBIA: Neurodegeneration with brain iron accumulation
PED: Paroxysmal exercise-induced dyskinesia
PKD: Paroxysmal kinesigenic dyskinesia
PNKD: Paroxysmal nonkinesigenic dyskinesia
RDP: Rapid-onset dystonia parkinsonism
SD: Spasmodic dysphonia
TBZ: Tetrabenazine
THC: Tetrahydrocannabinoid
U.S.: United States
VMAT2: Vesicular monoamine transporter 2
