# Correction to: Medical treatment of dystonia

**DOI:** 10.1186/s40734-018-0075-5

**Published:** 2018-11-16

**Authors:** Pichet Termsarasab, Thananan Thammongkolchai, Steven J. Frucht

**Affiliations:** 1 Movement Disorder Division, Department of Neurology, Icahn School of Medicine at Mount Sinai, New York, USA; 2 Department of Neurology, University Hospitals Cleveland Medical Center, Cleveland, USA

## Correction

Following publication of the original article [1], the authors reported that the videos referred to in their article were not accessible to readers. This was due to an error in the production workflow. The publisher apologises to the readers and the authors for the inconvenience.

The video files are provided in this Correction article as additional files.


Additional file 1: Video segment 1 demonstrates examples of dystonia mimics and psychogenic dystonia. (MP4 225047 kb)



Additional file 2: Video segment 2 demonstrates select examples of “don’t-miss” diagnoses. (MP4 133675 kb)



Additional file 3: Video segment 3 demonstrates example of patients with primary dystonia. (MP4 166317 kb)



Additional file 4: Video segment 4 demonstrates doparesponsive dystonia (DRD; Patients 1–2), one of the most crucial “don’t-miss” diagnoses in dystonia, and paroxysmal kinesigenic dyskinesia (Patients 3–5). (MP4 59281 kb)



Additional file 5: Video segment 5 demonstrates a variety of focal dystonias. (MP4 186066 kb)



Additional file 6: Video segment 6 demonstrates a woman with left hemidystonia. (MP4 68071 kb)



Additional file 7: Video segment 7 demonstrates a variety of tardive dystonias. (MP4 103680 kb)

